# Value-based healthcare implementation in the Netherlands: a quantitative analysis of multidisciplinary team performance

**DOI:** 10.1186/s12913-024-10712-x

**Published:** 2024-02-21

**Authors:** Henrike J. Westerink, Gijs Steinmann, Maarten Koomans, Michèle H. van der Kemp, Paul B. van der Nat

**Affiliations:** 1 Department of Value Improvement, St. Antonius Hospital, Koekoekslaan 1, 3430EM Nieuwegein, Nieuwegein, 3430 EM the Netherlands; 2 Scientific Center for Quality of Healthcare (IQ Health), Radboud University Medical Center, Geert Grooteplein Zuid 10, Nijmegen, 6525 GA the Netherlands; 3 Department of Health Services Research, Care and Public Health Research Institute, Faculty of Health, Medicine and Life Sciences, Maastricht University, Maastricht, the Netherlands; 4 Working group ‘Integrated Practice Units’, Linnean, Zeist, the Netherlands; 5 Value-Based Healthcare Strategy & Tactics, VDKMP, Amsterdam, the Netherlands

**Keywords:** Value-based healthcare, Multidisciplinary teams, Team performance

## Abstract

**Background:**

Many hospitals worldwide have set up multidisciplinary Value Improvement (VI) teams that use the Value-Based Health Care (VBHC) theory to improve patient value. However, it remains unclear what the level of VBHC implementation is within these teams. We therefore studied the current level of VBHC implementation in VI teams.

**Methods:**

A questionnaire was developed based on the strategic agenda for value transformation and real-world experiences with VBHC implementation. The questionnaire consisted of 21 questions, mapped to seven domains, and was sent out to 25 multidisciplinary VI teams. Median scores for individual questions (scale = 1–5) and average scores per domain were calculated.

**Results:**

One hundred forty VI team members completed the questionnaire. The overall average score is 3.49. The ‘culture and responsibility’ domain obtained the highest average score (µ = 4.11). The domain ‘measure and improve outcomes’ and the domain ‘multidisciplinary team’ obtained average scores that are slightly higher than the overall average (µ = 3.78 and µ = 3.76 respectively), and the domains ‘strategy and organizational policy,’ ‘collaboration and sharing,’ and ‘IT and data’ scored a little below the overall average (µ = 3.41, µ = 3.32, and µ = 3.29 respectively). The domain ‘costs and reimbursement’ obtained the lowest average score (µ = 2.42) of all domains, indicating that the implementation of this particular aspect of VBHC remains lagging behind.

**Conclusions:**

Our results indicate activity in each of the questionnaire domains. To bring VBHC implementation to the next level, more attention should be given to the financial aspects. Our questionnaire can be used in future studies to identify improvements or differences within VI teams.

**Supplementary Information:**

The online version contains supplementary material available at 10.1186/s12913-024-10712-x.

## Background

Worldwide, healthcare systems are faced with the challenge of rising healthcare costs and significant disparities in quality of care [1]. To address this challenge, Value-Based Health Care (VBHC) has been introduced with the overarching goal for healthcare systems to improve value for patients, where value is defined as the health outcomes that matter to patients divided by the costs needed to achieve them [2]. Since its introduction, VBHC has received significant attention across different countries and healthcare providers, with some scholars going as far as labelling it a “global megatrend” [3].

In 2013, Porter and Lee proposed a six-point strategic agenda for healthcare providers to guide VBHC implementation. Their “value agenda” proposes to: reorganize care into Integrated Practice Units (IPUs) around medical conditions; systematically measure outcomes and costs at the level of medical conditions; implement bundled payments for care cycles; integrate care delivery across separate facilities; expand the reach of high-value services; and build a supporting information technology platform [4]. In 2022, van der Nat identified that the strategic agenda had several shortcomings when it comes to guiding actual VBHC implementation, and thus suggested four complementary items; ‘set up value-based quality improvement; integrate value in patient communication; invest in a culture of value delivery; and build learning platforms for healthcare professionals’ [5]. Yet, despite the (extended) strategic agenda, recent reviews of the literature continue to reveal multiple studies that highlight challenging, complex, and often fragmented implementation processes [6, 7].

One of the main challenges regarding VBHC implementation concerns the transition towards IPUs. While Porter and colleagues advocate IPUs as the ultimate organizational structure for VBHC implementation in their value agenda [2, 4, 8], many hospitals maintain their traditional structures (with specialty-based budgets and lines of authority) and set up multidisciplinary Value Improvement (VI) teams around medical conditions between specialty units [9, 10]. These multidisciplinary VI teams are examples of a more incremental VBHC implementation strategy [9], which is used by different healthcare providers across multiple countries [6].

Although VBHC has received significant attention in terms of academic literature [6], a particular focus on the level of teams has thus far been lacking. Indeed, while many hospitals tend to implement VBHC at the level of medical conditions via multidisciplinary VI teams [6, 9], the majority of literature on VBHC implementation describes implementation at the organizational level [11–15]. Therefore, it remains unclear what the progress of VBHC implementation is at the level of VI teams and whether these teams are successful in the implementation of the different aspects of VBHC. Insight into VBHC implementation within VI teams is important to identify main challenges that lie ahead in the path towards more value for patients. In this paper, we therefore aim to provide insight into the progress of VBHC implementation at the level of VI teams, by surveying Dutch VI team members on their performance.

## Methods

### Questionnaire development

In order to measure the self-reported performance of multidisciplinary VI teams, a questionnaire was developed by a workgroup of the Linnean initiative. The Linnean initiative is a Dutch network that focusses on the acceleration of implementation of VBHC within the Dutch healthcare system [16] and has multiple workgroups, one of which focuses on IPUs. The 24 members of this IPU workgroup are experts in VBHC implementation and have hands-on experience with the transition towards organizing care around medical conditions [17]. A first draft of the questionnaire was developed by one of the co-authors and members of the IPU workgroup (MvdK). This draft was complemented with questions based on the Health Outcomes Management Evaluation model [14] and on what is known as the (extended) strategic agenda for value transformation [4, 5]. Consequently, the first draft was discussed with the members of the workgroup and refined based on their experiences and core literature on VBHC. Consensus on the questionnaire was reached after nine meetings, of which four meetings were held with a small group of delegates from the workgroup. In between these sessions, the questionnaire was piloted in a few VI teams. A few alterations were made after this pilot-testing, particularly regarding the answer options and length of text.

The developed questionnaire comprised 21 questions mapped to seven domains, i.e. ‘multidisciplinary teams’, ‘measure and improve outcomes’, ‘costs and reimbursements’, ‘collaboration and sharing’, ‘IT and data’, ‘culture and responsibility’, and ‘strategy and organizational policy’. Each domain consisted of three questions. Respondents received each question with two outer statements, ranging from low to high VBHC implementation, and were asked to rate their team within the two outer statements on a 5-point Likert-type scale. Examples of questions are:

- *‘To what extent are all relevant medical and support staff sufficiently represented in your team?’*

Where respondents were asked to rate their team within these two statements on a scale from 1 to 5:

- *1= The team is mono-disciplinary and/or mono-professional*
- *5= The team consists of all relevant medical staff, support staff, and management of all organizations in the full care cycle (this could be transmural)*

Or: *“To what extent a* *re the costs and reimbursements for the medical condition known?”*

Where respondents were asked to rate their team within these two statements on a scale from 1 to 5:

- *1= The costs and reimbursements for the medical condition are not known within the team*
- *5= The actual costs (in EUR) and reimbursements are known*

The workgroup drafted the questions and the two outer statements to be in line with the current state and focus of VBHC implementation within the Netherlands. See Additional file 1 for the full version of the questionnaire.

## Data collection

Between September and October 2021, we approached 35 project leaders and managers from multidisciplinary VI teams via the network of the co-authors by way of purposeful sampling aimed at obtaining data from a balanced variety of teams in terms of patient characteristics (e.g. type of medical condition), types of organization (e.g. general hospital, academic hospital, independent treatment center), and location within the Netherlands. In addition, teams were selected that have at least one year experience with VBHC, that are a representative team for the organisation, and consist of at least 5 team members, providing us with some assurance of the multidisciplinary character of the teams. Questionnaires were sent to project leaders and managers of VI teams via Google Forms, with the request to distribute the questionnaire to all VI team members of eligible teams, both healthcare professionals as well as (managerial) support staff. Questionnaires where only distributed after the project leader or manager had indicated their agreement to participate in the study.

## Analysis

Descriptive statistics were used. Median scores and interquartile ranges were calculated for individual questions with Microsoft Excel. In addition, numbers and percentages of missing answers at the level of individual questions were calculated. To assess the performance of multidisciplinary VI teams in each of the seven domains, average scores were calculated at the domain level, combining the results of the three underlying questions per domain. Furthermore, we categorized the teams into high-, average-, and low-scoring teams to study differences in VBHC implementation between these three categories (Additional file 2).

## Results

### Participants

Between September 2021 and November 2021 we surveyed 140 members out of 25 VI teams (Table 1). Ten of the approached teams were not included, mainly since these project leaders did not respond to our invitation. An average of 6 persons per team responded to the questionnaire, with a minimum of 1 and a maximum of 12 respondents per team.

**Table 1:** Respondent characteristics

Characteristics	Number
Total of participating teams	25
Type of hospital	
Top clinical teaching hospital	13 (52%)
Academic	6 (24%)
Independent Treatment Centre (ZBC)	3 (12%)
General hospital	3 (12%)
Medical condition/ patient group	
Arthrosis	1
Percutaneous coronary intervention	1
Birth care	2
Hip arthritis	2
Cerebrovascular accident	1
Head neck oncology	1
Turner syndrome	1
Kidney failure	2
Hand and wrist injuries	1
Prostate cancer	2
Knee injuries	1
Vestibular schwannoma	1
Trauma geriatrics	1
Breast cancer	2
Rheumatoid arthritis	2
Groin rupture	1
Cleft	1
Colorectal carcinoma	1
Pituitary adenoma	1

### Questionnaire results

**Fig. 1:**
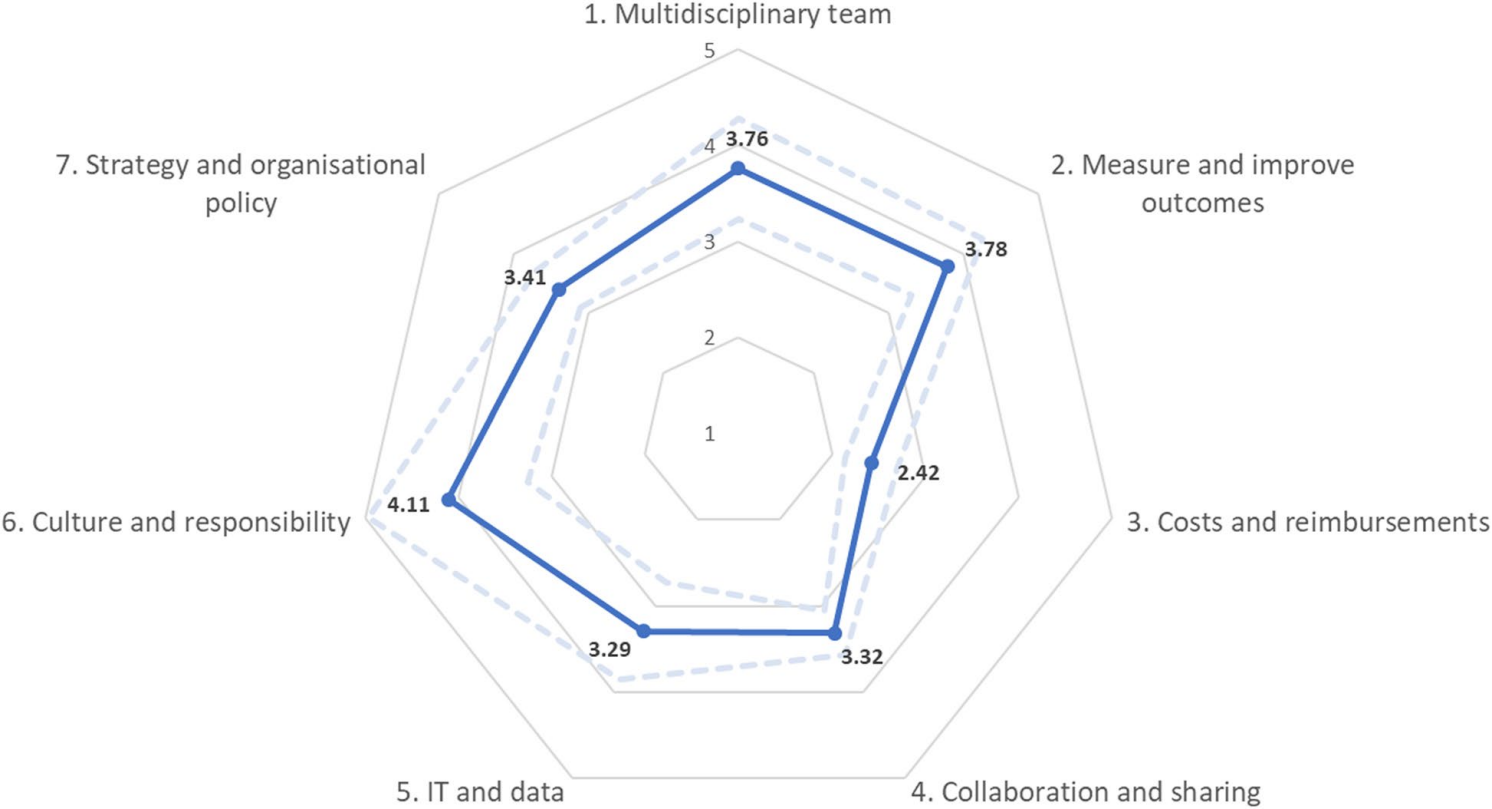
Radar diagram of average scores per domain of the questionnaire. Dotted line is the average plus or minus standard deviation

 The overall median score of all questions is four and the overall average score of all domains combined is 3.49. Table 2 shows the median scores and missing data for each of the subquestions. The majority of questions have an interquartile range of 1 or 2. Multiple questions from different domains have a high number of missing data, i.e. questions in the domains ‘costs and reimbursements’, ‘collaboration and sharing’, ‘IT and data’, and ‘strategy and organizational policy’. The missing scores are not related to specific teams. Average scores per domain are shown in Fig. 1.

**Table 2:**
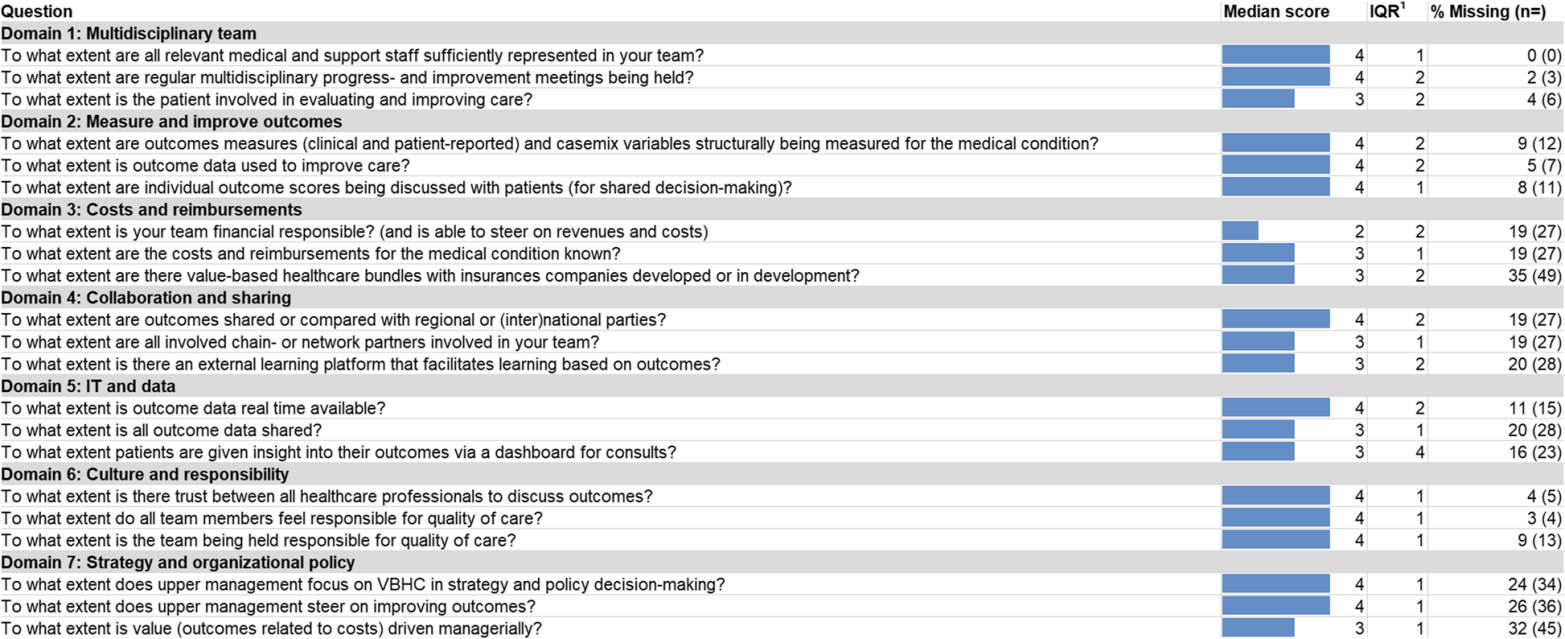
Questionnaire results

1= Interquartile range

### Domain 1: multidisciplinary team

Within the domain ‘multidisciplinary team’, median scores ranged between three and four. Respondents thus indicated that they perceive their VI teams to include close to all medical and supporting staff that are involved in their care cycle, and that their team has regular multidisciplinary meetings (Mdn = 4). The question on patient involvement, however, acquired a relatively lower score (Mdn = 3).

### Domain 2: measure and improve outcomes

Within this domain, all questions acquired a median score of four. In general, respondents indicated that their team is (structurally) involved in outcome measurement; that their team incorporates outcome data in efforts to improve healthcare delivery; and that outcome measurements of individual patients are discussed with them by healthcare professionals.

### Domain 3: costs and reimbursements

In this domain, median scores ranged between two and three. Generally, respondents indicated that they experienced relatively low financial responsibility and relatively low ability to steer on cost and revenue streams (Mdn = 2). The question on whether the costs and reimbursements for the medical condition are known, and the question regarding the presence and development of VBHC bundles both acquired a slightly higher score (Mdn = 3). Interestingly, for all three questions we found a relatively high number of missing data (ranging from *n* = 27–49), with the question on bundled payment showing the largest number of missing answers of all questions across domains (*n* = 49).

### Domain 4: collaboration and sharing

Questions in this domain acquired median scores between three and four. Respondents thus reported that they perceive their teams to share or compare outcome measurements with external parties (Mdn = 4). Both the question on the involvement of care chain relations and network partners in the team, as well as the question on external learning platforms, obtained somewhat lower scores (Mdn = 3). For all three questions in this domain, we found a relatively high number of missing data (ranging from *n* = 27–28).

### Domain 5: IT and data

Median scores within the ‘IT and data’ domain ranged between three and four. Respondents indicated that, in general, outcome data are real-time available (Mdn = 4). Regarding the question on sharing all outcome data, the respondents indicated a moderate level of sharing (Mdn = 3). The question on patients having insight into their outcome data via dashboards also obtained a median score of three. What stood out for this question, however, was the high variability of scores (IQR = 4). For all three questions in this domain, we found over 10% of missing answers (ranging from *n* = 15–28).

### Domain 6: culture and responsibility

All questions in this domain obtained a median score of four. Respondents thus indicated to experience (near) complete trust among their team members with regard to discussing outcomes; they also feel jointly responsible for quality of care that their team delivers. Moreover, respondents reported that their teams are (formally) held accountable for the quality of care of their care cycles.

### Domain 7: strategy and organizational policy

Within this domain, median scores ranged between three and four. Respondents indicated that VBHC is a focal point within upper management decision-making (Mdn = 4), which includes a managerial push to improve outcomes (Mdn = 4). The managerial push on patient value obtained a slightly lower score when (next to outcomes) costs are also taken into account (Mdn = 3). Overall, we found a relatively high number of missing data for this domain, with all three questions missing well over 20% of responses (ranging from *n* = 34–45).

### Comparison between domains

Taken together, the average score of all questions of all domains is 3.49. The ‘culture and responsibility’ domain obtained the highest average score (µ = 4.11). Respondents thus indicated that this aspect of VBHC implementation is progressing relatively well. Both the domain ‘measure and improve outcomes’ and the domain ‘multidisciplinary team’ obtained average scores that are slightly higher than the overall average (µ = 3.78 and µ = 3.76 respectively). The domains ‘strategy and organizational policy,’ ‘collaboration and sharing,’ and ‘IT and data’ scored a little below the overall average (µ = 3.41, µ = 3.32, and µ = 3.29 respectively). The domain ‘costs and reimbursement’ obtained the lowest average score (µ = 2.42) of all domains. With a score that is well below the overall average, respondents thus indicated that the implementation of this particular aspect of VBHC remains lagging behind within their teams.

When categorizing the teams into three categories, i.e. high-, medium-, or low-scoring teams, the data shows that high-scoring teams outscore the low-scoring teams on all domains, indicating that VI teams improve gradually in every domain, instead of excelling in one specific domain (Additional file 2).

## Discussion

This is the first study that provides an overview of the current level of VBHC implementation within multidisciplinary VI teams across multiple healthcare providers. To this end, we sent out a questionnaire that measures the self-reported performance of VI teams. Overall, our results show that VI teams are active in each of the questionnaire domains.

In the domain ‘multidisciplinary team’, respondents indicated that they have regular meetings with their VI team, involving close to all relevant medical and support staff. However, slightly lower scores were found for the question on patient participation within the VI teams. Yet, the importance of meaningful patient participation within VI teams had been pointed out by several studies, since patients can help to identify patient relevant outcomes and improvement initiatives [10, 11, 18, 19]. There is, however, a lack of guidance concerning patient participation within VBHC implementation [10], which leaves many VI teams struggling with the incorporation of patient participation in their team, and may at least partially explain the lower score for this particular question in our results.

Within the domain ‘measuring and improving outcomes’, the teams scored relatively high on all questions. This might be due to the fact that ‘measuring outcomes’ is often mentioned as a starting point for VBHC implementation [11]. Interestingly, respondents scored the use of outcome data to support shared decision-making relatively high, while the concept of shared decision-making was not part of the primary literature on VBHC. Over the years, shared decision-making has been accepted and embraced to be an important application of outcome data in healthcare [20, 21], and the high score in our study confirms this acknowledgement of shared decision-making being an integral part of VBHC in the Netherlands [22].

The domain ‘costs and reimbursements’ scored the lowest of all domains. This may well be due to the fact that VI teams – unlike Porter’s IPUs [2, 8] – maintain their traditional lines of funding, which results in little to no formally shared responsibility for financial aspects and cost-efficiency among team members. Furthermore, the question on the development of bundled payment contracts had the highest number of missing data, indicating that this question might be difficult to answer for VI team members. Value-based payment models are increasingly being adopted in the United States [23], but in the Netherlands the focus of VBHC has mainly been on improving care, and the cost aspect is often not taken into account [11]. In 2021, a large project was initiated in the Netherlands to stimulate the implementation of value-based payment models [24, 25]. At the team level, cost-driver indicators such as use of expensive medication are being utilized, but these only provide indirect insight into costs [9]. This little insight into the costs of care is striking, since rising healthcare costs is an often-mentioned reason for the implementation of VBHC.

Within the domain ‘collaboration and sharing’, respondents indicated that outcome data are shared externally, but that there is low involvement of chain and network partners in the teams. Furthermore, the questionnaire results imply an insufficiency when it comes to external learning platforms. Research has already shown that collaboration between different healthcare providers is a challenge in the current Dutch healthcare system, but that the development of a care chain with regional collaboration does improve both clinical and patient-reported outcomes [26]. Moreover, it is beneficial to collaborate with primary care partners, as they collect relevant outcome data that are part of VBHC standard outcome sets [27]. This suggests that VI teams should give more attention to collaboration with network partners over the full cycle of care to further improve value for patients.

Within the ‘IT and data’ domain, the question concerning real-time availability of outcome data had a high median score, which implies that outcome data are real-time available for healthcare professionals, and that PROMs are available in the personal health environment of the patient. Availability of outcome data towards the patient is an important aspect of patient empowerment and self-management, since this enables the patient to track their health status over time [28]. However, the lower score and high variability in scores of the question regarding availability and usage of dashboards to discuss outcome data with patients suggests that these data are not regularly being made visual, while this visualization of outcome data is an important step in order to use outcome data for shared decision-making [29].

Of the seven domains, the ‘culture and responsibility’ was the highest scoring one, indicating that there is trust among the team members to discuss outcomes, and that they feel and are being held accountable for the quality of care. The importance of formalized responsibility for outcome improvement has been stipulated in literature [13]. Interestingly, a previous study has shown that VI teams are often not fully responsible for quality and costs of care, since traditional responsibility lies at the level of departments [10]. The high scores within this domain suggest that steps have been made to further formalize responsibility for quality of care within VI teams, but the lower score of the question on financial responsibility (of domain 3) indicates that these steps have not yet been made for the financial aspects of VBHC. However, it is difficult to assign full financial responsibility to VI team members, since they often maintain traditional lines of responsibility and funding. Accordingly, improvements on this domain may well require a transition towards real IPUs, instead of multidisciplinary VI teams.

Lastly, questions within the domain ‘strategy and organizational policy’ had the lowest response rates, indicating that these questions were difficult to answer (perhaps respondents were not up-to-date on their organizational policy on VBHC). This could imply that there has mainly been a bottom-up approach for the set-up of VI teams. Meanwhile, the importance of strong leadership supportive of VBHC implementation has been highlighted several times in literature as shown by a literature review [30]. This suggests the need for improved organization-wide policies on VBHC and improved communication on these policies towards staff members [31].

### Limitations

We acknowledge some limitations in this study. First, we did not define all answer options (scores 2–4 on the Likert-scale) which resulted in difficulties with drawing a conclusion on what participants meant with these scores and could have influenced the results when respondents were confused by the two statements. We therefore recommend that the next version of the questionnaire defines all answer options, or uses a more traditional Likert scale (e.g. ranging from totally disagree-totally agree). Secondly, high scores in this questionnaire do not imply that VBHC implementation of these aspects is finished. VBHC is evolving, and questions and answer options on VBHC implementation therefore evolve with it. The questions of our questionnaire are in line with the current level of implementation of VBHC in the Netherlands. This is also the reason why not all aspects of Porter’s strategic agenda are included in the questionnaire (e.g. expand geographic reach), since these aspects have received little attention (so far) in the Netherlands. We therefore decided to not validate the questionnaire, because this validation might not be applicable for the next version. Thirdly, there might be a bias in respondents, since project leaders and managers might be incentivized to only forward the questionnaire to high-functioning multidisciplinary VI teams. However, we believe that we have reached an inclusive sample in terms of maturity of multidisciplinary VI teams, based on the different types of hospitals and variations in scores. Fourthly, we are unable to calculate a response rate, since we are unsure to how many team members the questionnaire was forwarded to by the project leaders/managers. Fifthly, some questions had a high number of missing data, indicating that these questions were considered to be difficult to answer, either because the respondents do not know the answer to this question for their team, because they did not consider these aspects as relevant for their team, or because they did not understand these questions. The low response rates could therefore have skewed the results, where in reality scores could be lower for several questions or domains.

## Conclusion

Overall, our results indicate activity in each of the VBHC implementation domains within multidisciplinary VI teams, with some variations between different domains. To bring VBHC implementation to the next level, more attention should be given to the financial aspects of VBHC, including financial responsibility. Furthermore, not only is there a need for more guidance on collaboration with patients in VI teams, there is also a need for increased collaboration with network partners over the full cycle of care. Future research should focus on these aspects of VBHC implementation, and what barriers and facilitators VI teams experience in these collaborations. Key inquiries include team awareness regarding these aspects as integral elements of VBHC, reasons for not initiating working on these aspects, and whether prioritizing to work on other aspects was an informed decision. Lastly, the results suggest that most VI teams are currently mainly set up via a bottom-up approach, and could benefit from improved organization-wide policies on VBHC. Future work should focus on these aspects in order to accelerate VBHC implementation. Our questionnaire can be used in these studies to identify improvements within these domains. Moreover, individual teams can use the questionnaire to measure their perceived level of VBHC implementation, and whether this differs between different team members.

### Supplementary Information

**Additional file 1.** Questionnaire including response options.

**Additional file 2.** Radar diagram of average scores per domain of the questionnaire when teams are categorized in high-, average-, or low-scoring teams.

## Data Availability

The datasets used and/or analysed during the current study are available from the corresponding author upon reasonable request.
