# Supplementary material for: Value-based healthcare implementation in the Netherlands: a quantitative analysis of multidisciplinary team performance
Source: BMC Health Serv Res. 2024 Feb 21;24:224. doi: 10.1186/s12913-024-10712-x (PMC10882801; doi:10.1186/s12913-024-10712-x)
Supplement: Supplementary file 1 — Additional file 1. Questionnaire including response options. [file 12913_2024_10712_MOESM1_ESM.docx]

# Appendix 1: Questionnaire

| **Question** | **Score 1** | **Score 5** |
| --- | --- | --- |
| **Domain 1: Multidisciplinary team** |  |  |
| To what extent are all relevant medical and support staff sufficiently represented in your team? | The team is mono-disciplinary and/or mono-professional | The team consists of all relevant medical staff, support staff, and management staff of all organizations in the full care cycle (this could be transmural) |
| To what extent are regular multidisciplinary progress- and improvement meetings being held? | These are not being held | There is structural and ad-hoc coordination |
| To what extent is the patient involved in evaluating and improving care? | The patient is not involved | The patient is a member of the multidisciplinary team and is present at the meetings and part of decision-making |
| **Domain 2: Measure and improve outcomes** |  |  |
| To what extent are outcomes measures (clinical and patient-reported) and case mix variables structurally being measured for the medical condition? | Outcomes (clinical and patient-reported) and case-mix variables are not structurally measured | A set of outcomes (clinical and patient-reported) and case-mix variables are structurally measured |
| To what extent is outcome data used to improve care? | Outcome data is not used for improvement projects | There is an improvement cycle. Outcome data is measured, improvement potential is identified, concrete goals are formulated, the improvement project is implemented, and evaluated to continue or stop |
| To what extent are individual outcome scores being discussed with patients (for shared decision-making)? | Individual outcome scores are not discussed with the patient | Individual outcome scores are always discussed with the patient (if they want to) and is fundamental for shared decision-making |
| **Domain 3: Costs and reimbursements** |  |  |
| To what extent is your team responsible for the financial aspects? | The team is not able to steer on cost and revenue streams | The team has full financial responsibility, including cost and revenue streams |
| To what extent are the costs and reimbursements for the medical condition known? | The costs and reimbursements for the medical condition are not known within the team | The actual costs (in EUR) and reimbursements are known |
| To what extent are there value-based healthcare bundles with insurances companies developed or in development? | There is no value-based healthcare bundle in development | A value-based healthcare bundle based on outcomes has been agreed on |
| **Domain 4: Collaboration and sharing** |  |  |
| To what extent are outcomes shared or compared with regional or (inter)national parties? | Outcomes are not being shared or compared with regional or (inter)national parties | Outcomes are being shared and compared with regional or (inter)national parties |
| To what extent are all involved chain- or network partners involved in your team? | There is no collaboration with chain- or network partners | The chain- and network partners are part of the team |
| To what extent is there an external learning environment that facilitates learning based on outcomes? | There is no learning environment | Outcomes are being shared in a joint improvement cycle and the different parties are part of everyone’s learning environment |
| **Domain 5: IT and data** |  |  |
| To what extent is outcome data real time available? | Outcome data is not digitally available | Outcome data is real-time available for healthcare professionals. PROMs are also available in the personal health environment of the patient |
| To what extent is outcome data shared? | Outcome data is shared at the team level | Outcome data is shared at the team level, organization level and externally |
| To what extent are the outcomes per patient made transparent via a dashboard for consults? | Outcome data is not being made visually per patient in a dashboard | Outcome data is being made visual per patient in a dashboard for the consultation in which individual outcomes can be compared to outcomes of patients with similar characteristics |
| **Domain 6: Culture and responsibility** |  |  |
| To what extent is there trust between all healthcare professionals to discuss outcomes? | There is no trust in our team | There is complete trust within the team |
| To what extent do all team members feel responsible for quality of care? | The team members only feel responsible for the quality of their personal care | All team members feel jointly responsible for the quality of care that the team delivers |
| To what extent is the team being held accountable for quality of care? | The team is not responsible for the quality of care that they deliver | The team is being held formally accountable for the quality of care over the full care cycle (including chain- and network partners). This is also captured in job descriptions (tasks, authorizations, and responsibilities) |
| **Domain 7: Strategy and organizational policy** |  |  |
| To what extent does higher management focus on VBHC in strategy and policy decision-making? | Value for the patient (outcomes and costs) does not play a role in strategy and policy decision-making | The patient value (outcomes and costs) is the most important criterion for every strategic and political decision |
| To what extent does higher management focus on improving outcomes? | There is only focus on process improvements, not on improving outcomes | Outcomes are being measured and concrete goals are formulated |
| To what extent is value (outcomes related to costs) driven managerially? | The costs and outcomes are not known at the managerial level | Costs are being measured and concrete goals for costs in relation to outcomes are formulated |
