# Supplementary material for: Value-based healthcare implementation in the Netherlands: a quantitative analysis of multidisciplinary team performance
Source: BMC Health Serv Res. 2024 Feb 21;24:224. doi: 10.1186/s12913-024-10712-x (PMC10882801; doi:10.1186/s12913-024-10712-x)
Supplement: Supplementary file 2 — Additional file 2. Radar diagram of average scores per domain of the questionnaire when teams are categorized in high-, average-, or low-scoring teams. [file 12913_2024_10712_MOESM2_ESM.docx]

# Appendix 2

**Figure 1| Radar diagram of average scores per domain of the questionnaire when teams are categorized in high-, average-, or low-scoring teams.**
